# Supplementary material for: Heat adaptation of phage T7 under an extended genetic code
Source: Virus Evol. 2021 Dec 1;7(2):veab100. doi: 10.1093/ve/veab100 (PMC8923235; doi:10.1093/ve/veab100)
Supplement: veab100_Supp [file veab100_supp.zip › Sequencing_Analysis.zip!/Sequencing_Analysis/A2/evidence/SNP_12.html]

BRESEQ :: Evidence


| Predicted mutation | | | | | | |
| --- | --- | --- | --- | --- | --- | --- |
| evidence | seq id | position | mutation | annotation | gene | description |
| RA | NC\_001604.1 | 29,697 | C→A | intergenic (–/–) | – / – | –/– |

| Read alignment evidence... | | | | | | | | | | | |
| --- | --- | --- | --- | --- | --- | --- | --- | --- | --- | --- | --- |
|  | seq id | position | | ref | new | freq | score (cons/poly) | reads | annotation | genes | product |
| \* | NC\_001604.1 | 29,697 | 0 | C | A | 87.7% | 523.1 / 101.9 | 379 | intergenic (–/–) | *–/–* | –/– |
| Reads supporting (aligned to +/- strand):  **ref** base C (20/26);  **new** base A (168/160);  **total** (191/188) | | | | | | | | | | | |
| Fisher's exact test for biased strand distribution *p*-value = 3.48e-01 | | | | | | | | | | | |
| Kolmogorov-Smirnov test that lower quality scores support variant *p*-value = 9.85e-01 | | | | | | | | | | | |

Only 100 of 379 total aligned reads displayed.  

`GTTCAGAATCAGATGAACGCATGGACGAAAGCTCAGGCCAAGGCTCTGGACGATTCCATGAAGTCAATGAACAAACTTGACGTAATCGACAAGCAATTCCAGAAGCGAATCAACGGTGAGTGGGTCTCAACGGATTTTAAGGATATGCCAGTCAACGAGAACACTGGTGAGTTCAAGCATAGCGATATGGTTAACTACGCCAATAAGAAGCTCGCTGAGATTGACAGTATGGACATTCCAGACGGTGCCAAGGATGCTATGAAGTTGAAGTACCTTCAAGCGGACTCTAAGGACGGAGCATTCCGTACAGCCATCGGAACCATGGT‑‑CACTGACGCTGGTCAAGAGTGGTCTGCCGCTGTGATTAACGGTAAGTTACCAGAACGAACCCCAGCTATGGATGCTCTGCGCAGAATCCGCAATGCTGACCCTCAGTTGATTGCTGCGCTATACCCAGACCAAGCTGAGCTATTCCTGACGATGGACATGATGGACAAGCAGGGTATTGACCCTCAGGTTATTCTTGATGCCGACCGACTGACTGTTAAGCGGTCCAAAGAGCAACGCTTTGAGGATGATAAAGCATTCGAGTCTGCAC  >  NC_001604.1/29399‑29993`  

                                                                                                                                                                                                                                                                                                          `|`                                                                                                                                                                                                                                                                                                            
`gTTCAGAATCAGATGAACGCATGGACGAAAGCTCAGGCCAAGGCTCTGGACGATTCCATGAAGTCAATGAACAAACTTGACGTAATCGACAAGCAATTCCAGAAGCGAATCAACGGTGAGTGGGTCTCAACGGATTTTAAGGATATGCCAGTCAACGAGAACACTGGTGAGTTCAAGCATAGCGATATGGTTAACTACGCCAATAAGAAGCTCGCTGAGATTGACAGTATGGACATTCCAGACGGTGCCAAGGATGCTATGAAGTTGAAGTACCTTCAAGCGGACTCTAAGGACGGAGAAt                                                                                                                                                                                                                                                                                                          >  1:93953/1‑301 (MQ=255)`  
    `aGAATCAGATGAACGCATGGACGAAAGCTCAGGCCAAGGCTCTGGACGATTCCATGAAGTCAATGAACAAACTTGACGTAATCGACAAGCAATTCCAGAAGCGAATCAACGGTGAGTGGGTCTCAACGGATTTTAAGGATATGCCAGTCAACGAGAACACTGGTGAGTTCAAGCATAGCGATATGGTTAACTACGCCAATAAGAAGCTCGCTGAGATTGACAGTATGGACATTCCAGACGGTGCCAAGGATGCTATGAAGTTGAAGTACCTTCAAGCGGACTCTAAGGACGGAGTATTCCg                                                                                                                                                                                                                                                                                                      >  1:572195/1‑301 (MQ=255)`  
      `aaTCAGATGAACGCATGGACGAAAGCTCAGGCCAAGGCTCTGGACGATTCCATGAAGTCAATGAACAAACTTGACGTAATCGACAAGCAATTCCAGAAGCGAATCAACGGTGAGTGGGTCTCAACGGATTTTAAGGATATGCCAGTCAACGAGAACACTGGTGAGTTCAAGCATAGCGATATGGTTAACTACGCCAATAAGAATCTCGCTGAGATTGACAGTATGGACATTCCAGACGGTGCCAAGGATGCTATGAAGTTGAAGTACCTTCAAGCGGACTCTAAGGACGGAGTATTCCgct                                                                                                                                                                                                                                                                                                    >  1:545248/1‑299 (MQ=255)`  
      `aaTCAGATGAACGCATGGACGAAAGCTAAGGCCAAGGCTCTGGACGATTCCATGAAGTCAATGAACAAACTTGACGTAATCGACAAGCAATTCCAGTAGCGAATCAACGGTGAGTGGGTCTCAACGGATTTTAAGGATATGCCAGTCAACGAGAACACTGGTGAGTTCAAGCATAGCGATATGGTTAACTACGCCAATAAGAAGCTCGCTGAGATTGACAGTATGGACATTCCAGACGGTGCCAAGGATGCTATGAAGTTGAAGTACCTTCAAGCGGACTCTAAGGACGGAGAATTCCGTa                                                                                                                                                                                                                                                                                                    >  1:361730/1‑301 (MQ=255)`  
       `aTCAGATGAACGCATGGACGAAAGCTCAGGCCAAGGCTCTGGACGATTCCATGAAGTCAATGAACAAACTTGACGTAATCGACAAGCAATTCCAGAAGCGAATCAACGGTGAGTGGGTCTCAACGGATTTTAAGGATATGCCAGTCAACGAGAACACTGGTGAGTTCAAGCATAGCGATATGGTTAACTACGCCAATAAGAAGCTCGCTGAGATTGACAGTATGGACATTCCAGACGGTGCCAAGGATGCTATGAAGTTGAAGTACCTTCAAGCGGACTCTAAGGACGGAGAATTCCATAc                                                                                                                                                                                                                                                                                                   <  1:737239/301‑1 (MQ=255)`  
       `aTCAGATGAACGCATGGACGAAAGCTCAGGCCAAGGCTCTGGACGATTCCATGAAGTCAATGAACAAACTTGACGTAATCGACAAGCAATTCCAGAAGCGAATCAACGGTGAGTGGGTCTAAACGGATTTTAAGGATATGCCAGTCAACGAGAACACTGGTGAGTTCAAGCATAGCGATATGGTTAACTACGCCAATAAGAAGCTCGCTGAGATTGACAGTATGGACATTCCAGACGGTGCCAAGGATGCTATGAAGTTGAAGTACCTTCAAGCGGACTCTAAGGACGGAGAATTCGGTAc                                                                                                                                                                                                                                                                                                   <  1:54141/301‑1 (MQ=255)`  
         `cAGATGAACGCATGGACGAAAGCTCAGGCAAAGGCTCTGGACGATTCCATGAAGTCAATGAACAAACTTGACGTAATCGACAAGCAATTCCAGAAGCGAATCAACGGTGAGTGGGTCTCAACGGATTTTAAGGATATGCCAGTCAACGAGAACACTGGTGAGTTCAAGCATAGCGATATGGTTAACTACGCCAATAAGAAGCTCGCTGAGATTGACAGTATGGACATTCCAGACGGTGCCAAGGATGCTATGAAGTTGAAGTACCTTCAAGCGGACTCTAAGGACGGAGAATTCCGTACAg                                                                                                                                                                                                                                                                                                 >  1:180374/1‑301 (MQ=255)`  
          `aGATGAACGCATGGACGAAAGCTCAGGCCAAGGCTCTGGACGATTCCATGAAGTCAATGAACAAACTTGACGTAATCGACAAGCAATTCCAGAAGCGAATCAACGGTGAGTGGGTCTCAACGGATTTTAAGGATATGCCAGTCAACGAGAACACTGGTGAGTTCAAGCATAGCGATATGGTTAACTACGCCAATAAGAAGCTCGCTGAGATTGACAGTATGGACATTCCAGACGGTGCCAAGGATGCTATGAAGTTGAAGTACCTTCAAGCGGACTCTAAGGACGGAGAATTCCGTACAGc                                                                                                                                                                                                                                                                                                <  1:513461/301‑1 (MQ=255)`  
                   `cATGGACGAAAGCTCAGGCCAAGGCTCTGGACGATTCCATGAAGTCAATGAACAAACTTGACGTAATCGACAAGCAATTCCAGAAGCGAATCAACGGTGAGTGGGTCTCAACGGATTTTAAGGATATGCCAGTCAACGAGAACACTGGTGAGTTCAAGCATAGCGATATGGTTAACTACGCCAATAAGAAGCTCGCTGAGATTGACAGTATGGACATTCCAGACGGTGCCAAGGATGCTATGAAGTTGAAGTACCATCAAGCGGACTCTAAGGACGGAGAATTCCGTACAGCCATCAGAAc                                                                                                                                                                                                                                                                                       <  1:42314/301‑1 (MQ=255)`  
                       `gACGAAAGCTCAGGCCAAGGCTCTGGACGATTCCATGAAGTCAATGAACAAACTTGACGTAATCGACAAGCAATTCCAGAAGCGAATCAACGGTTAGTGGGTCTCAACGGATTTTAAGGATATGCCAGTCAACGAGAACACTGGTGAGTTCAAGCATAGCGATATGGTTAACTACGCCAATAAGAAGCTCGTTGAGATTGACAGTATGGACATTCCAGACGGTGCCAAGGATGCTATGAAGTTGAAGTACCTTCAAGCGGACTCTAAGGACGGAGAATTCCGTACAGcctgtctcttataa                                                                                                                                                                                                                                                                                   >  1:236332/1‑289 (MQ=255)`  
                              `gCTCAGGCCAAGGCTCTGGACGATTCCATGAAGTCAATGAACAAACTTGACGTAATCGACAAGCTATTCCAGAAGCGAATCAACGGTGAGTGGGTCTCAACGGATTTTAAGGATATGCCAGTCAACGAGAACACTGGTGAGTTCAAGCATAGCGATATGGTTAACTACGCCAATAAGAAGCTCGCTGAGATTGACAGTATGGACATTCCAGACGGTGCCAAGGATGCTATGAAGTTGAAGTACCTTCAAGCGGACTCTAAGGACGGAGAATTCCGTACAGCCATCGGAACCATGGT‑‑CACTg                                                                                                                                                                                                                                                                          <  1:43711/301‑1 (MQ=255)`  
                              `gCTCAGGCCAAGGCTCTGGACGATTCCATGAAGTCAATGAACAAACTTGACGTAATCGACAAGCAATTCCAGAAGCGAATCAACGGTGAGTGGGTCTCAACGGATTTTAAGGATATGCCAGTCGACGAGAACACTGGTGAGTTCAAGCATAGCGATATGGTTAACTACGCCAATAAGAAGCTCGCTGAGATTTACAGTATGGACATTCCAGACGGTGCCAAGGATGCTATGAAGTTGAAGTACCTTCAAGCGGACTCTAAGGACGGAGAATTCCGTACAGCCATCGGAACCATGGT‑‑CACTg                                                                                                                                                                                                                                                                          >  1:374395/1‑301 (MQ=255)`  
                               `cTCAGGCAAAGACTCTGGACGATTCCATGAAGTCAATGAACAAACTTGACGTAATCGAAAAGCAATTCCAGAAGCGAATCAACGGTGAGTGGGTCTCAACGGATTTTAAGGATATGCCAGTCAACGAGAAAACTGGTGAGTTAAAGCATAGCGATATGGTTAACTACGCCAATAAGAAGCTCGCTGAGATTGACAGTATGGACATTCCAGACGGTGCCAAGGATGCTATGAAGTTGAAGTACCTTCAAGCGGACTCTAAGGACGGAGAATTCCGTACAGCCATCGGAACCATGGT‑‑CACTGa                                                                                                                                                                                                                                                                         <  1:385946/301‑1 (MQ=255)`  
                                            `cctGGTCGATTCCATGAAGTCAATGAACAAACTTGACGTAATCGACAAGCAATTCCAGAAGCGAATCAACGGTGAGAGGGTCTCAACGGATTTTAAGGATATGCCAGTCAACGAGAACACTGGTGAGTTCAAGCATAGCGATATGGTTAACTACGCCCATAAGAAGCTCGCTGAGATTGACAGTATGGACATTCCAGACGGTGCCAAGGATGCTATGAAGTTGAAGTACCTTCAAGCGGACTCTAAGGACGGAGAATTCCGTACAGCCATCGGAACCATGGT‑‑CACTGACGCTGGTCAagag                                                                                                                                                                                                                                                            <  1:339637/300‑1 (MQ=255)`  
                                            `aatgtgtataagagacaggTCAATGAACAAACTTGACGTAATCGACAAGCAATTCCAGAAGCGAATCAACGGTGAGTGGGTCTCAACGGATTTTAAGGATATGCCAGTCAACGAGAACACTGGTGAGTTCAAGCATAGCGATATGGTTAACTACGCCAATAAGAAGCTCGCTGAGATTGACAGTATGGACATTCCAGACGGTGCCAAGGATGCTATGAAGTTGAAGTACCTTCAAGCGGACTCTAAGGACGGAGAATTCCGTACAGCCATCGGAACCATGGT‑‑CACTGACGCTGGTCAagag                                                                                                                                                                                                                                                            <  1:260582/283‑1 (MQ=255)`  
                                               `ggACGATTCCATGAAGTCAATGAACAAACTTGACGTAATCGACAAGCAATTCCAGAAGCGAATCAACGGTGAGTGGGTCTCAACGGATTTTAAGGATATGCCAGTCAACGAGAACACTGGTGAGTTCAAGCATAGCGATATGGTTAACTACGCCAATAAGAAGCTCGCTGAGATTGACAGTATGGACATTCCAGACGGTGCCAAGGATGCTATGAAGTTGAAGTACCTTCAAGCGGACTCTAAGGACGGAGCATTCCGTACAGCCATCGGAACCATGGT‑‑CACTGACGCTGGTCAAGAGTgg                                                                                                                                                                                                                                                         >  1:442582/1‑301 (MQ=255)`  
                                                     `ttCCATTAAGTCAATGAACAAACTTGACGTAATCGACAAGCAATTCCAGAAGCGAATCAACGGTGAGTGGGTCTCAACGGATTTTAAGGATATGCCAGTCAACGAGAACACTGGTGAGTTCAAGCATAGCTATATGGTTAACTACGCCAATAAGAAGCTCGCTGAGATTGACAGTATGGACATTCCAGACGGTGCCAAGGATGCTATGAAGTTGAAGTACCTTCAAGCGGACTCTAAGGACGGAGAATTCCGTACAGCCATCGGAACCATGGT‑‑CACTGACGCTGGTCAAGAGTGGTCTGcc                                                                                                                                                                                                                                                   <  1:556181/301‑1 (MQ=255)`  
                                                           `aaaGTCAATGAACAAACTTGACGTAATCGACAAGCAATTCCAGAAGCGAATCAACGGTGAGTGGGTCTCAACGGATTTTAAGGATATGCCAGTCAACGAGAACACTGGTGAGTTCAAGCATAGCGATATGGTTAACTACGCCAATAAGAAGCTCGCTGAGATTGATAGTATGGACATTCCAGACGGTGCCAAGGATGCTATGAAGTTGAAGTACCTTCAAGCGGACTCTAAGGACGGAGAATTCCGTACAGCCATCGGAACCATGGT‑‑CACTGACGCTGGTCAAGAGTGGTCTGCCGCtgtg                                                                                                                                                                                                                                             <  1:330854/300‑1 (MQ=255)`  
                                                                   `tGAACAAACTTGACGTAATCGACAAGCAATTCCAGAAGCGAATCAACGGTGAGTGGGTCTCAACGGATTTTAAGGATATGCCAGTCAACGAGAACACTGGTGAGTTCAAGCATAGCGATATGGTTAACTACGCCAATAAGAAGCTCGCTGAGATTGACAGTATGGACATTCCAGACGGTGCCAAGGATGCTATGAAGTTGAAGTACCTTCAAGCGGACTCTAAGGACGGAGAATTCCGTACAGCCATCGGAACCATGGT‑‑CACTGACGCTGGTCactgtctcttatacacatctccgagccc                                                                                                                                                                                                                                     >  1:157371/1‑274 (MQ=255)`  
                                                                   `gtgggctcggagatgtgtataagagacagttCCAGAAGCTAATCAACGGTGAGTGGGTCTCAACGGATTTTAAGGATATGCCAGTCAACGAGAACACTGGTGAGTTAAAGCATAGCGATATGGTTAACTACGCCAATAAGAAGCTCGCTGAGATTGACAGTATGGACATTCCAGACGGTGCCAAGGATGCTATGAAGTTGAAGTACCTTCAAGCGGACTCTAAGGACAGAGAATTCAGTACAGCCATCGGAACCATGGT‑‑CACTGACGCTGGTCAAGAGTGGTCTGCCGCTGTGATTAACgg                                                                                                                                                                                                                                     <  1:451335/272‑1 (MQ=255)`  
                                                                        `agatgtgtataagagacagaGCAATTCCAGAAGCGAATCAACGGTGAGTGGGTCTCAACGGATTTTAAGGATATGCCAGTCAACGAGAACACTGGTGAGTTCAAGCATAGCGATATGGTTAACTACGCCAATAAGAAGCTCGCTTAGATTGACAGTATGGACATTCCAGACGGTTCCAAGGATGCTATGAAGTTGAAGTACCTTCAAGCGGACTCTAAGGACGGAGCATTCCGTACAGCCATCGGAACCATGGT‑‑CACTGACGCTGGTCAATAGTGGTCTGCCGCTGTGATTAACGGTAAGt                                                                                                                                                                                                                                <  1:619838/282‑1 (MQ=255)`  
                                                                         `aaCTTGACGTAATCGACAAGCAATTCCAGAAGCGAATCAACGGTGAGTGGGTCTCAACGGATTTTAAGGATATGCCAGTCAACGAGAACACTGGTGAGTTCAAGCATAGCGATATGGTTAACTACGCCAATAAGAAGCTCGCTGAGATTGACAGTATGGACATTCCAGACGGTGCCAAGGATGCTATGAAGTTGAAGTACCTTCAAGCGGACTCTAAGGACTGAGAATTCCGTACAGCCATCGGAACCATGGT‑‑CACTGACGCTGGTCAAGAGTGGTCTGCCGCTGTGATTAACGGTAAGtt                                                                                                                                                                                                                               <  1:595011/301‑1 (MQ=255)`  
                                                                         `aaCTTGACGTAATCGACAAGCAATTCCAGAAGCGAATCAACGGTGAGTGGGTCTCAACGGATTTTAAGGATATGCCAGTCAACGAGAACACTGGTGAGTTCAAGCATAGCGATATGGTTAACTACGCCAATAAGAAGCTCGCTGAGATTGACAGTATGGACATTCCAGACGGTGCCAAGGATGCTATGAAGTTGAAGTACCTTCAAGCGGACTCTAAGGACGGAGAATTCCGTACAGCCATCGGAACCATGGT‑‑CACTGACGCTGGTCAAGAGTGGTCTGCCGCTGTGATTAACGGTAAGtt                                                                                                                                                                                                                               <  1:230709/301‑1 (MQ=255)`  
                                                                                     `tCGACAAGCAATTCCAGAAGCGAATCAACGGTGAGTGGGTCTCAACGGATTTTAAGGATATGCCAGTCAACGAGAACACTGGTGAGTTCAAGCATAGCGATATGGTTAACTACGCCAATAAGAAGCTCGCTGAGATTGACAGTATGGACATTCCAGACGGTGCCAAGGATGCTATGAAGTTGAAGTACCTTCAAGCGGACTCTAAGGACGGAGAATTCCGTACAGCCATCGGAACCATGGT‑‑CACTGACGCTGGTCAAGAGTGGTCTGCCGCTGTGATTAACGGtctgtctcttatacacat                                                                                                                                                                                                                   >  1:315434/1‑284 (MQ=255)`  
                                                                                      `cGACAAGCAATTCCAGAAGCGAATCAACGGTGAGTGGGTCTCAACGGATTTTAAGGATATGCCAGTCAACGAGAACACTGGTGAGTTCAAGCATAGCGATATGGTTAACTACGCCAATAAGAAGCTCGCTGAGATTGACAGTATGGACATTCCAGACGGTGCCAAGGATGCTATGAAGTTGAAGTACCTTCAAGCTGACTCTAAGGACGGAGAATTCCGTACAGCCATCGGAACCATGGT‑‑CACTGACGCTGGTCAAGAGTGGTCTGCCGCTGTTATTAACGGTAAGTTACCAGAACGAAcc                                                                                                                                                                                                                  <  1:198405/301‑1 (MQ=255)`  
                                                                                      `cGACAAGCAATTCCAGAAGCGAATCAACGGTGAGTGGGTCTCAACGGATTTTAAGGATATGCCAGTCAACGAGAACACTGGTGAGTTCAAGCATAGCGATATGGTTAACTACGCCAATAAGAAGCTCGCTGAGATTGACAGTATGGACATTCCAGACGGTGCCAAGGATGCTATGAAGTTGAAGTACCTTCAAGCGGACTCTAAGGACGGAGAATTCCGTACAGCCATCGGAACCATGGT‑‑CACTGACGCTGGTCAAGAGTGGTCTGCCGCTGTGATTAACGATAAGTTAccctgtctctta                                                                                                                                                                                                                  >  1:63530/1‑291 (MQ=255)`  
                                                                                             `cAATTCCAGAAGCGAATCAACGGTGAGTGGGTCTCAACGGATTTTAAGGATATGCCAGTCAACGAGAACACTGGTGAGTTCAAGCATAGCGATATGGTTAACTACGCCAATAAGAAGCTCTCTGAGATTGACAGTATGGACATTCCAGACGGTGCCAAGGATGCTATGAAGTTGAAGTACCTTCAAGCGGACTCTAAGGACGGAGCATTCCGTACAGCCATCGGAACCATGGT‑‑CACTGACGCTGGTCAAGAGTGGTCTGCCGCTGTGATTAACGGTAAGTTACCAGAACGAACACCAGCTa                                                                                                                                                                                                           >  1:577508/1‑301 (MQ=255)`  
                                                                                                 `gtgtataagagacagaCGGTGAGTGGGTCTCAACGGATTTTAAGGATATGCCAGTCAACGAGAACACTGGTGAGTTCAAGCATAGCGATATGGTTAACTACGCCAATAAGAAGCTCGCTGAGATTGACAGTATGGACATTCCAGACGGTGCCAAGGATGCTATGAAGTTGAAGTACCTTCAAGCGGACTCTAAGGACGTAGAATTCCGTACAGTCATCGGAACCATGGT‑‑CACTGACGCTGGTCAAGAGTGGTCTGCCGCTGTGATTAACGGTAAGTTACCAGAACGAACCCCAGCTATGGa                                                                                                                                                                                                       <  1:559942/286‑1 (MQ=255)`  
                                                                                                  `ccAGAAGCGAATAAACGGTGAGTGGGTATCAACGGATTTTAAGGATATGCCAGTCAACGAGAACACTGGTGAGTTCAAGCATAGCGATATGGTTAACTACGCCAATAAGAAGCTCGCTGAGATTGACAGTATGGACATTCCAGACGGTGCCAAGGATGCTATGAAGTTGAAGTACCTTCAAGCGGACTCTAAGGACGGAGAATTCCGTACAGCCATCGGAACCATGGT‑‑CACTGACGCTGGTCAAGAGTGGTCTGCCGCTGTGATTAACGGTAAGTTACCAGAACGAACCCCAGCTATGGAt                                                                                                                                                                                                      <  1:274461/301‑1 (MQ=255)`  
                                                                                                    `aGAAGCGAATCAACGGTGAGTGGGTCTCAACGGATTTTAAGGATATGCCAGTCAACGAGAACACTGGTGAGTTCAAGCATAGCGATATGGTTAACTACGCCAATAAGAAGCTCGCTGAGATTGACAGTATGGACATTCCAGATGGTGCCAAGGATGCTATGAAGTTGAAGTACCTTCAAGCGGACTCTAAGGACGGAGAATTCCGTACAGCCATCGGAACCATGGT‑‑CACTGACGCTGGTCAAGAGTGGTCTGCCGCTGTGATTAACGGTAAGTTACCAGAACGAACCCCAGCTATGGATGc                                                                                                                                                                                                    <  1:145004/301‑1 (MQ=255)`  
                                                                                                        `gCGAATCAACGGTGAGTGGGTCTCAACGGATTTGAAGGATATGCCAGTCAACGAGAACACTGGTGAGTTCAAGCATAGCGATATGGTTAACTACGCCAATAAGAAGCTCGCTGAGATTGACAGTATGGACATTCCAGACGGTGCCAAGGATGCTATGAAGTTGAAGTACCTTCAAGCGGACTCTAAGGACGGAGAATTCCGTACAGCCATCGGAACCATGGT‑‑CACTGACGCTGGTCAAGAGTGGTCTGCCGCTGTGATTAACGGTAAGTTACCAGAACGAACCCCAGCTATGGATGCTCTg                                                                                                                                                                                                <  1:716907/301‑1 (MQ=255)`  
                                                                                                                     `gAGTGGGTCTCAACGGATTTTAAGGATATGCCAGTCAACTAGAACACTGGTGAGTTTAAGCATAGCGATATGGTTAACTACGCCAATAAGAAGCTCGCTTAGATTGACAGTATGTACATTCCAGACGGTGCCAAGGATGCTATGAAGTTGAAGTACCTTCAAGCGGACTCTAAGGACGTAGAATTCCGTACAGCCATCGGAACCATGGT‑‑CACTGACGCTGGTCAAGAGTGGTCTGCCGCTGTGATTAACGGTAAGTTACCAGAACGAACCCCAGCTATGGATGCTCTGCGCAGAATCCGCa                                                                                                                                                                                   >  1:646154/1‑301 (MQ=255)`  
                                                                                                                     `gAGTGGGTCTCAACGGATTTTAAGGATATGCCAGTCAACGAGAACACTGGTGAGTTCATGCATATCGATATGGTTAACTACGCCAATAAGAAGCTCGCTGAGATTGACAGTATGGACATTCCAGACGGTGCCAAGGATGCTATGAAGTTGAAGTACCTTCAAGCGGACTCTAAGGACGGAGAATTCCGTACAGCCATCGGAACCATGGT‑‑CACTGACGCTGGTCAAGAGTGGTCTGCCGCTGTGATTAACGGTAAGTTACCAGAACGAACCCCAGCTATGGATGCTCTGCGCAGAATctgtc                                                                                                                                                                                   >  1:808258/1‑297 (MQ=255)`  
                                                                                                                     `gAGTGGGTCTCAACGGATTTTAAGGATATGCCAGTCAACGAGAACACTGGTGAGTTCAAGCATAGCGATATGGTTAACTACGCCAATAAGAAGCTAGCTGAGATTGACAGTATGGACATTCCAGACGGTGCCAAGGATGCTATGAAGTTGAAGTACCTTCAAGCGGACTCTAAGGACGGAGAATTCCGTACAGCCATCGGAACCATGGT‑‑CACTGACGCTGGTCAAGAGTGGTCTGCCGCTGTGATTAACGGTAAGTTACCAGAACGAACCCAAGCTATGGATGCTCTGCGCAGAATCCGCa                                                                                                                                                                                   >  1:246627/1‑301 (MQ=255)`  
                                                                                                                         `gggTCTCAACGGATTTTAAGGATATGCCAGTCAACGAGAACACTGGTGAGTTCAAGCATAGCGATATGGTTAACTACGCCAATAAGAAGCTCGCTGAGATTGACAGTATGGACATTCCAGACGGTGCCAAGGATGCTATGAAGTTGAAGTACCTTCAAGCGGACTCTAAGGACGGAGCATTCCGTACAGCCATCGGAACCATGGT‑‑CACTGACGCTGGTCAAGAGTGGTCTGCCGCTGTGATTAACGGTAAGTTACCAGAACGAACCCCAGCTATGGATGCTCTGCGCAGAATCCGCACTGc                                                                                                                                                                               >  1:750791/1‑301 (MQ=255)`  
                                                                                                                                      `ttttAAGGATATGCCAGTCAACGAGAACACTGGTGAGTTCAAGCATAGCGATATGGTTAACTACGCCAATAAGAAGCTCGCTGAGATTGACAGTATGGACATTCCAGACGGTGCCAAGGATGCTATGAAGTTGAAGTACCTTCAAGCGGACTCTAATGACGTAGAATTCCGTACAGCCATCGGAACCATGGTCACACTGACGCTGGTCAAGAGTGGTCTGCCGCTGTGATTAACGGTAAGTTACCAGAACGAACCCCAGCTATGGATGCTCTGCGCAGAATCCGCAATGCTGACCCTCAGt                                                                                                                                                                    >  1:797886/1‑301 (MQ=255)`  
                                                                                                                                        `cttggagatgtgtataagagacagacacTGGTGAGTTCAAGCATAGCGATATGGTTAACTACGCCAATAAGAAGCTCGCTGAGATTGACAGTATGGACATTCCAGACGGTGCCAAGGATGCTATGAAGTTGAAGTACCTTCAAGCGGACTCTAAGGACGGAGAATTCCGTACAGCCATCGGAACCATGGT‑‑CACTGACGCTGGTCAAGAGTGGTCTGCCGCTGTGATTAACGGTAAGTTACCAGAACGAACCCCAGCTATGTATGCTCTGCGCAGAATCCGCAATGCTGACCCTCAGTTGAt                                                                                                                                                                <  1:320171/277‑1 (MQ=255)`  
                                                                                                                                           `aGGATATGCCAGTCAACGAGAACACTGGTGAGTTCAAGCATAGCGATATGGTTAACTACGCCAATAAGAAGCTCGCTGAGATTGACAGTATGGACATTCCAGACGGTGCCAAGGATGCTATGAAGTTGAAGTACCTTCAAGCGGACTCTAAGGACGGAGAATTCCGTACAGCCATCGGAACCATGGT‑‑CACTGACGCTGGTCAAGAGTGGTCTGCCGCTGTGATTAACGGTAAGTTACCAGAATGAACCCCAGCTATGGATGCTCTGCGCAGAATCCGCAATGCTGACCCTCAGTTGATtgc                                                                                                                                                             <  1:532523/301‑1 (MQ=255)`  
                                                                                                                                            `ggATATGCCAGTCAACGAGAACACTGGTGAGTTCAAGCATAGCGATATGGTTAACTACGCCAATAAGAAGCTCGCTGAGATTGACAGTATGGACATTCCAGATGGTGCCAAGGATGCTATGAAGTTGAAGTACCTTCAAGCGGACTCTAAGGACGGAGAATTCCGTACAGCCATCGGAACCATGGT‑‑CACTGACGCTGGTCAAGAGTGGTCTGCCGCTGTGATTAACGGTAAGTTACCATAACGAACCCCAGCTATGGATGCTCTGCGCAGAATCCACAATGCTGACCCTCAGTTGATtgct                                                                                                                                                            >  1:612225/1‑301 (MQ=255)`  
                                                                                                                                            `ggATATGCCAGTCAACGAGAACACTGGTGAGTTCAAGCATAGCGATATGGTTAACTACGCCAATAAGAAGCTCGCTGAGATTGACAGTATGGACATTCCAGACGGTGCCAAGGATGCTATGAAGTTGAAGTACCTTCAAGCGGACTCTAAGGACGGAGAATTCCGTACAGCCATCGGAACCATGGT‑‑CACTGACGATGGTCAAGAGTGGTCTGCCGCTGTGATTAACAGTAAGTTACCAGAACGAACCCCAGCTATGGATGCTCTGCACAGAATCCGCAATGCTGACCCTCAGTTGATtgct                                                                                                                                                            >  1:216370/1‑301 (MQ=255)`  
                                                                                                                                              `atatGCCAGTCAACGAGAACACTGGTGAGTTCAAGCATAGCGATATGGTTAACTACGCCAATAAGAAGCTCGCTGAGATTGACAGTATGGACATTCCAGACGGTGCCAAGGATGCTATGAAGTTGAAGTACCTTCAAGCGGACTCTAAGGACGTAGCATTCCGTACAGCCATCGGAACCATGGT‑‑CACTGACGCTGGTCAAGAGTGGTCTGCCGCTGTGATTAATGGTAAGTTACCAGAACGAACCCCATCTATGGATGCTCTGCGCAGAATCCGCAATGCTGACCCTCAGTTGATtgctgc                                                                                                                                                          >  1:126287/1‑301 (MQ=255)`  
                                                                                                                                                     `ggagatgtgtataagagacaggTTCAAGCATAGCGATATGGTTAACTACGCCAATAAGAAGCTCGCTGAGATTGACAGTATGGACATTCCAGACGGTGCCAAGGATGCTATGAAGTTGAAGTACCTTCAAGCGGACTCTAAGGACGGAGAATTCCGTACAGCCATCGGAACCATGGT‑‑CACTGACGCTGGTCAAGAGTGGTCTGCCGCTGTGATTAACGGTAAGTTACCAGAACGAACCCCAGCTATGGATGCTCTGCGCAGAATCCGCAATTCTGACCCTCAGTTGATTGCTGCGCTATAc                                                                                                                                                   <  1:652882/280‑1 (MQ=255)`  
                                                                                                                                                     `agacagcGAGAACACTGGTGAGTTCAAGCATAGCGATATGGTTAACTACGCCAATAAGAAGCTCGCTGAGATTGACAGTATGGACATTCCAGACGGTGCCAAGGATGCTATGAAGTTGAAGTACCTTCAAGCGGACTCTAAGGACGGAGAATTCCGTACAGCCATCGGAACCATGGT‑‑CACTGACGCTGGTCAAGAGTTGTCTGCCGCTGTGATTAACGGTAAGTTACCAGAACGAACCCCAGCTATGGATGCTCTGCGCAGAATCCGCAATGCTGACCCTCAGTTGATTGCTGCGCTATAc                                                                                                                                                   <  1:566882/295‑1 (MQ=255)`  
                                                                                                                                                      `gTCAACGAGAACACTGGTGAGTTCAAGCATAGCGATATGGTTAACTACGCCAATAAGAAGCTCGCTGAGATTGACAGTATGGACATTCCAGACGGTGCCAAGGATGCTATGAAGTTGAAGTACCTTCAAGCGGACTCTAAGGACGGAGAATTCCGTACAGCCATCGGAACCATGGT‑‑CACTGACGCTGGTCAAGAGTGGTCTGCCGCTGTGATTAACGGTAAGTTACCAGAACGAACCCCAGCTATGGATGCTCTGCGCAGAATACGCAATGCTGACCCTCAGTTGATTGCTGCGCTATAcc                                                                                                                                                  >  1:663589/1‑301 (MQ=255)`  
                                                                                                                                                           `gtgtataagagacaggTTCAAGCATAGCGATATGGTTAACTACGCCAATAAGAAGCTCGCTTAGATTGACAGTATGGACATTCCAGACGGTGCCAAGGATGCTATGAAGTTGAAGTACCTTCAAGCGGACTCTAAGGGCGGAGCATTCCGTACAGCCATCGGAACAATGGT‑‑CACTGACGCTGGTCAAGAGTGGTCTGCCGCTGTGATTAACGGTAAGTTACCAGAACGAACCCCAGCTATGGATGCTCTGCGCAGAATCCGCAATGCTGACCCTCAGTTGATTGCTGCGCTATACCCAGAc                                                                                                                                             <  1:390498/286‑1 (MQ=255)`  
                                                                                                                                                            `gagaACACTGGTGAGTTCAAGCATAGCGATATGGTTAACTACGCCAATAAGAAGCTCGCTGAGATTGACAGTATGGACATTCCATACGGTGCCAAGGATGCTATGAAGTTGAAGTACCTTCAAGCGGACTCTAAGGACTGAGAATTCCGTACAGCCATCGTAACCATGGT‑‑CACTGACGCTGGTCAAGAGTGGTCTGCCGCTGTGATTAATGGTAAGTTACCAGAACGAACCCCAGCTATGGATGCTCTGCGCAGAATCCGCAATGCTGACCCTCAGTTGATTGCTGCACTATACCAAGAcc                                                                                                                                            <  1:302699/301‑1 (MQ=255)`  
                                                                                                                                                            `gagaACACTGGTGAGTTCAAGCATAGCGATATGGTTAACTACGCAAATAAGAAGCTCGCTGAGATTGACAGTATGGACATTCCAGACGGTGCCAAGGATGCTATGAAGTTGAAGTACCTTCAAGCGGACTCTAAGGACGGAGAATTCCGTACAGCCATCAGAACCATGGT‑‑CACTGACGCTGGTCAAGAGTGGTCTGCCGCTGTGATTAACGGTAAGTTACCAGAACGAACCACAGCTATGGATGCTCTGCGCAGAATCCGCAATGCTGACCCTCAGTTGATTGCTGCGCTATACCCAGAcc                                                                                                                                            <  1:288987/301‑1 (MQ=255)`  
                                                                                                                                                                `acacTGGTGAGTTCAAGCATAGCGATATGGTTAACTACGCCAATAAGAAGCTCGCTTAGATTGACAGTATGGACATTCCAGACGGTGCCAAGGATGCTATGAAGTTGAAGTACCTTCAAGCGGACTCTAAGGACGGAGAATTCCGTACAGCCATCGGAACCATGGT‑‑CACTGACGCTGGTCAAGAGTGGTCTGCCGCTGTGATTAACGGTAAGTTACCAGAACGAACCCCAGCTATGGATGCTCTGCGCAGAATCCGCAATGCTGACCCTCAGTTGATTGCTGCGCTATACCAAGACCAAGc                                                                                                                                        <  1:283889/301‑1 (MQ=255)`  
                                                                                                                                                                 `cacTGGTGAGTTCAAGCAAAGCGATATGGTTAACTACGCCAATAAGAAGCTCGCTGAGATTGACAGTATGGACATTCCAGACGGTGCCAAGGATGCTATGAAGTTGAAGTACCTTCAAGCGGACTCTAAGGACGGAGAATTCCGTACAGCCATCGGAACCATGGT‑‑CACTGACGCTGGTCAAGAGTGGTATGCCGCTGTGATTAACGGTAAGTTACCAGAACGAACCCCAGCTATGGATGCTCTGCGCAGAATCCGCAATGCTGACCCTCAGTTGATTGCTGCGCTATACCCAGACCAAGCt                                                                                                                                       <  1:702735/301‑1 (MQ=255)`  
                                                                                                                                                                     `ggTGAGTTCAAGCATAGCGATATGGTTAACTACGCCAATAAGAAGCTCGCTGAGATTGACAGTATGGACATTCCAGACGGTGCCAAGGATGCTATGAAGTTGAAGTACCTTCAAGCGGACTCTAAGGACGGAGAATTCCGTACAGCCATCGGAACCATGGT‑‑CACTGACGCTGGTCAAGAGTGGTCTGCCGCTGTGATTAACGGTAAGTTACCAGAACGAACCCCAGCTATGGATGCTCTGCGCAGAATCCGCAATGCTGACCCTCAGTTGATTGCTGCGCTATACCCAGACCAAGCTgctg                                                                                                                                   >  1:683522/1‑298 (MQ=255)`  
                                                                                                                                                                          `gTTCAAGCATAGCGATATGGTTAACTACGCCAATAAGAAGCTCGCTGAGATTGACAGTATGGACATTCCAGACTGTGCCAAGGATGCTATGAAGTTGAAGTACCTTCAAGCGGACTCTAAGGACGGAGAATTCCGTACAGCCATCGGAACCATGGT‑‑CACTGACGCTGGTCAAGAGTGGTCTGCCGCTGTGATTAACGGTAAGTTACCAGAACGAACCCCAGCTATGGATGCTCTGCGCAGAATCCGCAATGCTGACCCTCAGTTGATTGCTGCGCTATACCCAGACCAAGCTGAGCTATTc                                                                                                                              >  1:669875/1‑301 (MQ=255)`  
                                                                                                                                                                           `taagagacaggCGATATGGTTAACTACGCCAATAAGAAGCTCGCTGAGATTGACAGTATGGACATTACAGACGGTGCCAAGGATGCTATGAAGTTGAAGTACCTTCAAGCGGACTCTAAGGACGGAGAATTCCGTACAGCCATCGGAACCATGGT‑‑CACTGACGCTGGTCAAGAGTGGTCTGCCGCTGTGATTAACGGTAAGTTACCAGAACGAACCCCAGCTATGGATGCTCTGCGCAGAATCCGCAATGCTGACCCTCAGTTGATTGCTGCGCTATACCCAGACCAAGCTGAGCTATTcc                                                                                                                             <  1:241198/291‑1 (MQ=255)`  
                                                                                                                                                                             `cAAGCATAGCGATATGGTTAACTACGCCAATAAGAAGCTCGCTGAGATTGACAGTATGGACATTCCAGACGGTGCCAAGGATGCTATGAAGTTGAAGTACCTTCAAGCGGACTCTAAGGACGGCGAATTCCGTACAGCCATCGGAACCATGGT‑‑CACTGACGCTGGTCACGAGTGGTATACCGCTGTGATTAACGGTAAGTTACCAGAACGAACCCCAGCTATGGATGATCTGCGCATAATCCGCAATGCTGACCCTCAGTTGATTGCAGCGCTATCACCAGACCAAGCTTAGCtcatccct                                                                                                                           >  1:254927/1‑294 (MQ=255)`  
                                                                                                                                                                               `gtataagagacagggTTAACTACGCCAATAAGAAGCTCGCTGAGATTGACAGTATGGACATTCCAGACGGTGCCAAGGATGCTATGAAGTTGAAGTACCTTCAAGCGGACTCTAAGGACGGAGAATTCCGTACAGCCATCGGAACCATGGT‑‑CACTGACGCTGGTCAAGAGTGGTCTGCCGCTGTGATTAACGGTAAGTTACCAGAACGAACCCCAGCTATGGATGCTCTGCGCAGAATCCGCAATGCTGACCCTCAGTTGATTGCTGCGCTATACCCAGACCAAGCTGAGCTATTCCTGAc                                                                                                                         <  1:686429/288‑1 (MQ=255)`  
                                                                                                                                                                                     `gCGATATGGTTAACTACGCCAATAAGAAGCTCGCTGAGATTGACAGTATGGACATTCCAGACGGTGCCAAGGATGCTATGAAGTTGAAGTACCTTCAAGCGGACTCTAAGGACGGAGAATTCCGTACAGCCATCGGAACCATGGT‑‑CACTGACGCTGGTCAAGAGTGGTCTGCCGCTGTGATTAACGGTAAGTTACCAGAACGAACCCCAGCTATGGATGCTCTGCGCAGAATCCGCAATGCTGACCCTCAGTTGATTGCTGCGCTATACCCAGAcctgtctcttatacacatctccgagcc                                                                                                                   >  1:51005/1‑276 (MQ=255)`  
                                                                                                                                                                                      `cGATATGGTTAACTACGCCAATAAGAAGCTCGCTGAGATTGACAGTATGGACATTCCAGACGGTGCCAAGGATGCTATGAAGTTGAAGTACCTTCAAGCGGACTCTAAGGGCGGAGCATTCCGTACAGCCATCGGAACCATGGT‑‑CACTGACGCTGGTCAAGAGTGGTCTGCCGCTGTGATTAACGGCAAGTTACCAGAACGAACCCCAGCTATGGATGCTCTGCGCAGAATCCGCAATGCTGACCCTCAGTTGATTGCTGCGCTATACCCAGACCAAGCTGAGCTATTCCTGACGATGGAc                                                                                                                  >  1:631971/1‑301 (MQ=255)`  
                                                                                                                                                                                            `cgtgggctcggagatgtgtataagagacaggaTTGACAGTATGGACATTCCAGACGGTGCCAAGGATGCTATGAAGTTGAAGTACCTTCAAGCGGACTCTAAGGACGTAGAATTCCGTACAGCCATCGGAACCATGGT‑‑CACTGACGCTGGTCAAGAGTGGTCTGCCGCTGTGATTAACGGTAAGTTACCAGAACGAACCCCAGCTATGGATGCTCTGCGCAGAATCCGCAATGCTGACCCTCAGTTGATTGCTGCGCTATACCCAGACCAAGCTGAGCTATTCCTGACGATGGACatgatg                                                                                                            <  1:699068/271‑1 (MQ=255)`  
                                                                                                                                                                                               `tAACTACGCCAATAAGAAGCTCGCTGAGATTGACAGTATGGACATTCCAGACGGTGCCAAGGATGCTATGAAGTTGAAGTACCTTCAAGCGGACTCTAAGGACGGATAATTCCGTACAGCCATCGGAACCATGGT‑‑CACTGACGCTGGTCAAGAGTGGTCTGCCGCTGTGATTAACGGTAAGTTACCAGAACGAACCCCAGCTATGGATGCTCTGCGCATAATCCGCAATGCTGACCCTCAGTTGATTGCTGCGCTATACCCAGACCAAGCTGAGCTATTCCTTACGATGGACATGATGGAc                                                                                                         <  1:322567/301‑1 (MQ=255)`  
                                                                                                                                                                                               `aaaCTACGCCAATAAGAAGCTCGCTGAGATTGACAGTATGGACATTCCAGACGGTGCCAAGGATGCTATGAAGTTGAAGTACCTTCAAGCGGACTCTAAGGACGGAGAATTCCGTACAGCCATCGGAACCATGGT‑‑CACTGACGCTGGTCAAGAGTGGTCTGCCGCTGTGATTAACGGTAAGTTACCAGAACGAACCCCAGCTATGGATGCTCTGCGCAGAATCCGCAATGCTGACCCTCAGTTGATTGCTGCGCTATACCCAGACCAAGCTGAGCTATTCCTGACGATGGACATGATGGAc                                                                                                         <  1:169891/300‑1 (MQ=255)`  
                                                                                                                                                                                                   `tACGCCAATAAGAAGCTCGCTGAGATTGACAGTATGGACAGTCCAGACGGTGCCAAGGATGCTAAGAAGTTGAAGTACCTTCAAGCGGACTCTAAGGACGGAGAATTCCGTACAGCCATCGGAACCATGGT‑‑CACTGACGCTGGTCAAGAGTGGTCTGCCGCTGTGATTAACGGTAAGTTACCAGAACGAACCCCAGCTATGGATGCTCTGCGCAGAATCCGAAATGCTGACGCTCAGTTGATTGCTGCGCTATACCCAGACCAAGCTGAGCTATTCCTGACGATGGACATGATGGACAAGc                                                                                                     <  1:468214/301‑1 (MQ=255)`  
                                                                                                                                                                                                       `gatgtgtaaagagacaggagaTTGACAGTATTGACATTCCAGACGGTGCCAAGGATGCTATGAAGTTGAAGTACCTTCAAGCGGACTCTAAGGATGGAGAATTCCGTACAGCCATCGGAACCATGGT‑‑CACTGACGCTGGTCAAGAGTTGTCGTCCGCTGTGATTAACGGTAAGTTACCAGAACGAACCCCAGCTATGGATGCTCTGCGCAGAATCCGCAATGCTGACCCTCAGTTGATTGCTGCGCTATACCCAGACCAAGCTGAGCTATTCCTGACGATGGACATGATGGACAAGCAggg                                                                                                 <  1:24296/284‑1 (MQ=255)`  
                                                                                                                                                                                                              `gaagCTCGCTGAGATTGACAGTATGGACATTCCAGACGGTGCCAAGGATGCTATGAAGTTGAAGTACCTTCAAGCGGACTCTAAGGACGGAGAATTCCGTACAGCCATCGGAACCATGGT‑‑CACTGACGCTGGTCAAGAGTGGTCTGCCGCTGTGATTAACGGTAAGTTACCAGAACGAACCCCAGCTATGGATGCTCTGCGCAGAATCCGCAATGCTGACCCTCAGTTGATTGCTGCGCTATACCCAGACCAAGCTGAGCTATTCCTGACGATGGACATGATGGACAAGTAGGGTATTGAc                                                                                          <  1:349036/301‑1 (MQ=255)`  
                                                                                                                                                                                                              `gaagCTCGCTGAGATTGACAGTATGGACATTCCAGACGGTGCCAAGGATGCTATGAAGTTGAAGTACCTTCAAGCGGACTCTAAGGACGGAGAATTCCGTACAGCCATCGGAACCATGGT‑‑CACTGACGCTGGTCAAGAGTGGTCTGCCGCTGTGATTAACGGTAAGTTACCAGAACGAACCCCAGCTATGGATGCTCTGCGCAGAATCCGCAATGCTGACCCTCAGTTGATTGCTGCGCTATACCCAGACCAAGCTGAGCTATTCCTGACGATGGACATGATGGACAAGCAGGGTATTGAc                                                                                          <  1:135212/301‑1 (MQ=255)`  
                                                                                                                                                                                                               `aagCTCGCTGAGATTGACAGTATGGACATTCCAGACGGTGCCAAGGATGCTATGAAGTTGAAGTACCTTCAAGCGGACTCTAAGGACGGAGAATTCCGTACAGCCATCGGAACCATGGT‑‑CACTGACGCTGGTCAAGAGTGGTCTGCCGCTGTGATTAACGGTAAGTTACCAGAACGAACCCCAGCTATGGATGCTATGCGCAGAATCCGCAATGCTGACCCTCAGTTGATTGCTGCGCTATACCCAGACCAAGCTGAGCTATTCCTGACGATGGACATGATGGACAAGCAGGGTATTGAcc                                                                                         <  1:604896/301‑1 (MQ=255)`  
                                                                                                                                                                                                                 `gCTCGCTGAGATTGACAGTATGGACATTCCAGACGGTGCCAAGGATGCTATGAAGTTGAAGTACCTTCAAGCGGACTCTAAGGACGGAGAATTCCGTACAGCCATCGGAACCATGGT‑‑CACTGACGCTGGTCAAGAGTGGTCTGCCGCTGTGATTAACGGTAAGTTACCAGAACGAACCCCAGCTATGGATGCTCTGCGCAGAATCCGCAATGCTGACCCTCAGTTGATTGCTGCACTATACCCAGACCAAGCTGAGCTATTCCTGACGATGGACATGATGGAcctgtctcttatacacatc                                                                                       >  1:744991/1‑283 (MQ=255)`  
                                                                                                                                                                                                                 `gCTCGCTGAGATTGACAGTATGGACAGTCCAGACGGTGCCAAGGATGCTATGAAGTTGAAGTACCTTCAAGCGGACTCTAAGGACGGAGAATTCCGTACAGCCATCGGAACCATGGT‑‑CACTGACGCTGGTCAAGAGTGGTCTGCCGCTGTGATTAACGGTAAGTTACCAGAACGAACCCCAGCTATGGATGCTCTGCGCAGAATCCGCAATGCTGACCCTCAGTTGATTGCTGCGCTATACCCAGACCAAGCTGAGCTATTCCTGACGATGGACATGATGGACAAGCAGGGTATTGAACCt                                                                                       <  1:665542/301‑1 (MQ=255)`  
                                                                                                                                                                                                                    `cGCTGAGATTGACAGTATGGACATTCCAGACGGTGCCAAGGATGCTATGAAGTTGAAGTACCTTCAAGCGGACTCTAAGGACGGAGAATTCCGTACAGCCATCGGAACCATTGT‑‑CACTGACGCTGGTCAAGAGTGGTCTGCCGCTGTGATTAACGGTAAGTTACCAGAACGAACCCCAGCTATGGATGCTCTGCTCAGAATCCGCAATGCTGACCCTCAGTTGATTGCTGCGCTATACCCAGACCAAGCTGAGCTATTCCTGACGATGGACATGATGGACAAGCAGGGTATTGACCCTCAg                                                                                    >  1:116650/1‑301 (MQ=255)`  
                                                                                                                                                                                                                          `gaTTGACAGTATGGACATTACAGACGGTGCCAAGGATGCTATGAAGTTGAAGTACCTTCAAGCGGACTCTAAGGACGGAGAATTCCGTACAGCCATCGGAACCATGGT‑‑CACTGACGCTGGTCAAGAGTGGTCTGCCGCTGTGATTAACGGTAAGTTACCAGAACGAACCCAAGCTATGGATGCTCTGCGCAGAATCCGCAATGCTGACCCTCAGTTGATTGCTGCGCTATACCCAGACCAAGCTGAGCTATTCCTGACGATGGACATGCTGGACAAGCAGGGTATTGACCCCCAGGTTCtt                                                                              >  1:463632/1‑301 (MQ=255)`  
                                                                                                                                                                                                                            `gtGACAGTATGGACATTCCAGACGGTGCCAAGGATGCGATGAAGTTGAAGTACCTTCAAGCGGACTCTAAGGGCGGAGCATTCCGTACAGCCATCGGAACCATGGT‑‑AACTGACGCTGGTCAAGAGTGGTCTGCCGCTGTGATTAACGGTAAGTTACCAGAACGAACCCCAGCTATGGATGCTCTGCGCAGAATCCGCAATGCTGACCCTCAGTTGATTGCTGCGCTATACCCAGACCAAGCTGAGCTATTCCTGACGATGGACATGATGGACAAGCAGGGTATTGACCCTCAGGTTATTCt                                                                            <  1:49603/300‑1 (MQ=255)`  
                                                                                                                                                                                                                                  `gTATGGACATTCCAGACGGTGCCAAGGATGCTATGAAGTTGAAGTACCTTCAAGCGGACTCTAGGGACGGAGAATTCCGTACAGCCATCGGAACCATGGT‑‑CACTGACGCTGGTCAAGAGTGGTCTGCCGCTGTGATTAACGGTAAGTTACCAGAACGAACCCAAGCTATGGATGCTCTGCGCAGAATCCGCAATGCTGACCCTCAGTTGATTGCTGCGCTATACCCAGACCAAGCTGAGCTATTCATGACGATGGACATGATGGACAAGCAGGGTATTGACCCTCAGGTTATTCTTGATGc                                                                      >  1:101112/1‑301 (MQ=255)`  
                                                                                                                                                                                                                                  `gTATGGACATTCCAGACGGTGCCAAGGATGCTATGAAGTTGAAGTACCTTCAAGCGGACTCTAAGGACGGAGCATTCCGTACAGCCATCGGAACCATGGT‑‑CACTGACGCTGGTCAAGAGTGGTCTGCCGCTGTGATTAACGGTAAGTTACCAGAACGAACCCCAGCTATGGATGCTCTGCGCAGAATCCGCAATGCTGACCCTCAGTTGATTGCTGAGCTATACCCAGACCAAGCTGAGCTATTCCTGACGATGGACATGATGGACAAGCAGGGTATTGACCCTCAggctgtctcttatac                                                                      >  1:529714/1‑288 (MQ=255)`  
                                                                                                                                                                                                                                  `gTATGGACAGTCCAGACGGTGCCAAGGATGCTATGAAGTTGAAGTACCTTCAAGCGGACTCTAAGGACGGAGAATTCCGTACAGCCATCGGAACCATGGT‑‑CACTGACGCTGGTCAAGAGTGGTCTGCCGCTGTGATTAACGGTAAGTTACCAGAACGAACCCCAGCTATGGATGCTCTGCGCAGAATCCGCAATGCTGACCCTCAGTTGATTGCTGCGCTATACCCAGACCAAGCTGAGCTATTCCTGACGATGGACATGATGGACAAGCAGGGTATTGACCCTCAGGTTATTCTTGATGc                                                                      <  1:41242/301‑1 (MQ=255)`  
                                                                                                                                                                                                                                           `ttCCAGACGGTGCCAAGGATGCTATGAAGTTGAAGTACCTTCGAGCGGACTCTAAGGACGGAGAATTCCGTACAGCCATCGGAACCATGGT‑‑CACTGACGCTGGTCAAGAGTGGTCTGCCGCTGTGATTAACGGTAAGTTACAAGAACGAACCCCAGCTATGGATGCTCTGCGCAGAATCCGCAATGCTGACCCTCAGTTGATTGCTGCGCTATACCCAGACCAAGCTGAGCTATTCCTGACGATGGACATGATGGACAAGCAGGGTATTGACCCTCAGGTTATTCTTGATGCCGACCgact                                                             >  1:507417/1‑301 (MQ=255)`  
                                                                                                                                                                                                                                           `ttCCAGACGGTGCCAAGGATGCTATGAAGTTGAAGTACCTTCAAGCGGACTCTAAGGACAGAGAATTCCGTACAGCCATCGGAACCATGGT‑‑CACTGACGCTGGTCAAGAGTGGTCTGCCGCTGTGATTAACGGTAAGTTACCAGAACGAACCCCAGCTATGGATGCTCTGCGCAGAATCCGCAATGCTGACCCTCAGTTGATTGCTGCGCTATACCCAGACCAAGCTGAGCTATTCCTGACGATGGACATGATGGACAAGCAGGGTATTGACCATCAGGTTATTCTTGATGCCGACCgact                                                             >  1:617084/1‑301 (MQ=255)`  
                                                                                                                                                                                                                                               `aGATGGTGCCAAGGATGCTATGAAGTTGAAGTACCTTCAAGCGGACTCTAAGGACGGAGCATTCCGTACAGCCATCGGAACAATGGT‑‑CACTGACGCTGGTCAAGAGTGGTCTGCCGCTGTGATTAACGGTAAGTTACCAGAACGACCCCCAGCTATGGATGCTCTGCGCAGAATCCGCAATGCTGACCCTAAGTTGATTGCTGCGCTATACCCAGACCAAGCTGAGCTATTCCTGACGATGGACATGATGGACAAGCAGGGTATTGACCCTCAGGTTATTCTTGATGCCGACCgactgact                                                         >  1:22187/1‑301 (MQ=255)`  
                                                                                                                                                                                                                                                    `gTGCCAAGGATGCTATGAAGTTGAAGTACCTTCAAGCGGACTCTAAGGACGGAGAATTCCGTACAGCCATCGGAACCATGGT‑‑CACTGACGCTGGTCAAGAGTGGTCTGCCGCTGTGATTAACTGTAAGTTACCAGAACGAACCCCAGCTATGGATGCTCTGCGCAGAATCCGCAATGCTGACCCTCAGTTGATTGCTGCGCTATACCCAGACCAAGCTGAGCTATTCCTGACGATTGACATGATGGACAAGCAGGGTATTGACCCTCAGGTTATTCTTGATGCCGACCGACTGACTGTTaa                                                    >  1:638251/1‑301 (MQ=255)`  
                                                                                                                                                                                                                                                    `gTGCCAAGGATGCTATGAAGTTGAAGTACCTTCAAGCGGACTCTAAGGACGGAGAATTCCGTACAGCCATCGGAACCATGGT‑‑CACTGACGCTGGTCAAGAGTGGTCTGCCGCTGTGATTAACGGTAAGTTACCAGAACGAACCCCAGCTATGGATGCTCTGCGCAAAATCCGCAATGCTGACCCTCAGTTGATTGCTGCGCTATACCCAGACCAAGCTGAGCTATTCCTGACGATGGACATGATGGACAATCAGGGTATTGACCCTCAGGTTATTCTTGATGCCGACCGACTGACTGTTaa                                                    >  1:33535/1‑301 (MQ=255)`  
                                                                                                                                                                                                                                                      `ggtgggctcggagatgtgtataagagacagcAAGTGGACTCTAAGGACGGAGAATTCCGTACAGCCATCGGAACCATGGT‑‑CACTGACGCTGGTCAAGAGTGGTCTGCCGCTGTGATTAACGGTAAGTTACCAGAACGAACCCCAGCTATGGATGCTATGCGCAGAATCCGCAATGCTGACCCTCAGTTGATTGCTGCGCTATACCCAGACCAAGCTGAGCTATTCCTGACGATGGACATGATGGACAAGCAGGGTATTGACCCTCAGGTTATTCTTGATGCCGACCGACTGACTGTTAAGc                                                  <  1:132324/271‑1 (MQ=255)`  
                                                                                                                                                                                                                                                      `ggctcggagatgtgtataagagaccgccTTCAAGCGGACTCTAAGGACGGACAATTCCGTACAGCCATCGGAACCATGGT‑‑CACTGACGCTGGTAAAGAGTGGTCTGCCGCTGTGATTAACGGTAAGTTACCAGAACGAACCACAGCTATGGATGCTCTGCGCAGAATCCGCAATGCTGACCCGCAGTTGATTGCTGCGCTAAACCCAGACCAAGCTGAGCTATTCCTGACGATGGACATGATGGACAAGCAGGGTATTGACCATCAGGTTATTCTTGATGCCGACCGACTGACTGTTAAGc                                                  <  1:400629/275‑1 (MQ=255)`  
                                                                                                                                                                                                                                                      `gCCAATGATTCTATGAAGTTGAAGTCCCTTCAAGCGGACTCTAAGGACGGAGAATTCCGTACAGCCATCGTAACCATGGT‑‑CACTGACGCTGGTCAAGAGTGGTCTGCCGCTGTGATTAACGGTAAGTTACCATAACGAACCCCAGCTATGGATGCTCTGCGCAGAATCCGCAATGCTGACCCTCAGTTTATTGCTGCGCTATACCCAGACCAAGCTGAGCTATTCCTGACGATGGACATGATGGACAAGCAGGGTATTGACCCTCAGGTTATTCTTGATGCCTACCGACTGACTGTTAAGc                                                  <  1:486700/301‑1 (MQ=255)`  
                                                                                                                                                                                                                                                        `cAAGGATGCTATGAAGTTGAAGTACCTTCAAGCGGACTCTAAGGGCGGAGCATTCCATACAGCCATCGGAACCATGGT‑‑CACTGACGCTGGTCAAGAGTGGTCTGCCGCTGTGATTAACGGTAAGTTACCAGAACGAACCCCAGCTATGGATGCTATGCGCAGAATCCGCAATGCTGACCCTCAGTTGATTGCTGCGCTATACCCAGACCAAGCTGAGCTATTCCTGACGATGGACATGATGGACAAGCAGGGTATTGACCCTCAGGTTATTCTTGATGCCGACCAACTGACTGTTAAGCgg                                                <  1:522804/301‑1 (MQ=255)`  
                                                                                                                                                                                                                                                        `cAAGGATGCTATGAAGTTGAAGTACCTTCAAGCGGACTCTAAGGACGTAGAATTCCGTACAGCCATCGGAACCATGGT‑‑CACTGACGCTGGTCAAGAGTGGTCTGCCGCTGTGATTAACGGTAAGTTACCAGAACGAACCCCAGCTATGGATGCTCTGCGCAGAATCCGCAATGCTGACCCTCAGTTGATTGCTGCGCTATACCCAGACCAAGCTGAGCTATTCCTGACGATGGACATGATGGACAAGCAGGGTATTGACCCTCAGGTTATTCTTGATGCCTACCGACTGACTGTTAAGCgg                                                <  1:268192/301‑1 (MQ=255)`  
                                                                                                                                                                                                                                                           `ggATGCTATGAAGTTGAAGTACCTTCAAGCGGACTCTAAGGACGGAGAATTCCGTACAGCCATCGGAACCATGGT‑‑CACTGACGCTGGTCAAGAGTGGTCTGCAGCTGTGATTAACGGTAAGTTACCAGAACGAACCCCAGCTATGGATGATCTGCGCAGAATCCGCAATGCTGACCCTCAGTTGATTGCTGCGCTATACCCAGACCAAGCTGAGCTATTCATGACGATGGACATGATGGACAAGCAGGGTATTGACCCTCAGGTTATTCTTGATGCCGACCGACTGACTGTTAAGCGGTcc                                             >  1:62496/1‑301 (MQ=255)`  
                                                                                                                                                                                                                                                            `ggtGCTATGAAGTTGAAGTACCTTCAAGCGGACTCTAAGGACGGAGAATTCCGTACAGCCATCGGAACCATGGT‑‑CACTGACGCTGGTCAAGAGTGGTCTGCCGCTGTGATTAACGGTAAGTTACCAGAACGAACCCCAGCTATGGATACTCTGCGCAGAATCAGCAATGCTGACCCTCAGTTGATTGCTGCGCTATACCCAGACCAAGCTGAGCTATTCCTGACGATGGACATGATGGCCAACCAGGGTATTGACTCTCCGGTTATTCTTGATGCCGACCgactgacctgtctcttatact                                            >  1:536468/3‑287 (MQ=255)`  
                                                                                                                                                                                                                                                               `gCTATGAAGTTGAAGTACCTTCAAGCGGACTCTAAGGACTGAGAATTCCGTACAGCCATCGGAACCATGGT‑‑CACTGACGCTGGTCAAGAGTGGTCTGCCGCTGTGATTAACGGTAAGTTACCAGAACGAACCCCAGCTATGGATGCTCTGCGCAGAATCCGCAATGCTGACCCTCAGTTGATTGCTGCGCTATACCCAGACCAAGCTGAGCTATTCCTGACGATGGACATGATGTACAAGCAGGGTATTGACCCTCAGGTTATTCTTGATTCCGACCGACTGCCTGTTAAGCGGTCCaaaa                                         >  1:756077/1‑300 (MQ=255)`  
                                                                                                                                                                                                                                                                `gagacaggttgaagtACCTTCAAGCGGACTCTAATGACGGAGCATTCCGTACAGCCATCGGAACCATTGT‑‑CACTGACGCTGGTCAAGAGTGGTCTGCCGCTGTGATTAACGGTAAGTTACCAGAACGAACCCCAGCTATGGATGCTCTGCGCAGAATCCGCAATGCTGACCCTCAGTTGATTGCTGCGCTATACCCAGACCAAGCTGAGCTATTCCTGACGATGGACATGATGGACAAGCAGGGTATTGACCCTCAGGTTATTCTTGATGCCGACCGACTGACTGTTAAGCGGTCCAAaga                                        <  1:581595/294‑1 (MQ=255)`  
                                                                                                                                                                                                                                                                `agacagagttgaagtACCTTCAAGCGGACTCTAAGGACGGAGCATTCCGTACAGCCATCGGAACTATGGT‑‑CACTGACGCTGGTCAAGAGTGGTCTGCCGCTGTGATTAACGGTAAGTTACCAGAACGAACCCCAGCTATGGATGCTCTGCGCAGAATCCGCAATGCTGACCCTCAGTTGATTGCTGCGCTATACCCAGACCAAGCTGAGCTATTCCTGACGATGGACATGATGTACAAGCAGGGTATTGACCCTCAGGTTATTCTTGATGCCGACCGACTGACTGTTAAGCGGTCCAAaga                                        <  1:793044/295‑1 (MQ=255)`  
                                                                                                                                                                                                                                                                     `aagttgaagtACCTTCAAGCGGACTCTAAGGACGGAGAATTCCGTACAGCCATCGGAACCATGGT‑‑CACTGACGCTGGTCAAGAGTGGTCTGCCGCTGTGATTAACGGTAAGTTACCAGAACGAACCCCAGCTATGGATGCTCTGCGCAGAATCCGCAATGCTGACCCTCAGTTGATTGCTGCGCTATACCCAGACCAAGCTGAGCTATTCCTGACGATGGACATGATGGACAAGCAGGGTATTGACCCTCAGGTTATTCTTGATGCCGACCGACTGACTGTTAAGcctgtctcttatacac                                   >  1:724141/1‑286 (MQ=255)`  
                                                                                                                                                                                                                                                                       `gttgacctACCTTCATGCGGACGCTAAGGACGGAGATTTCCCTACAGCCATCGGAACCATGGT‑‑CACTGACGCTGGTCAAGAGTGATCTGCCTCTGTGATTAACGGTAAGTTACCAGAACGAACCCCAGCTATGGATGCTCTGCGCAGAATCCGCAATGCTGACCCTCAGTTGATTGCTGCGCTATACCCAGACCAAGCTGAGCTATTCCTGACGATGGACATGATGGACAAGCAGGGTATTTACCCTCAGGTTATTCTTGATGCCTACCGACTGACTGTTAAGCGGTCCAAAGAGCAACGc                                 <  1:583817/301‑1 (MQ=255)`  
                                                                                                                                                                                                                                                                         `tgaagtACCTTCAAGCGGACTCTAAGGACGGAGAATTCCGTACAGCCATCGGAACCATGGT‑‑CACTGACGCTGGTCAAGAGTGGTCTGCCGCTGTGATTAACGGTAAGTTACCAGAACGAACCCCAGCTATGGATGCTCTGCGCAGAATCCGCAATGCTGACCCTCAGTTGATTGCTGCGCTATACCCAGACCAAGCTGAGCTATTCCTGACGATGGACATGATTGACAAGCAGGGTATTTACCCTCAGGTTATTCTTGATGCCGACCGACTGACTGTTAAGCGGTCCAAAGAGCAACGCtt                               <  1:824554/301‑1 (MQ=255)`  
                                                                                                                                                                                                                                                                             `gtACCTTCAAGCGGACTCTAAGGACGGAGCATTCCGTACAGCCATCGGAACCATGGT‑‑CACTGACGCTGGTCAAGAGTGGTCTGCCGCTGTGATTAACGGTAAGTTACCAGAACGAACCCCAGCTATGGATGCTCTGCGCAGAATCCGCAATGCTGACCCTCAGTTGATTGCTGCGCTATACCCAGACCAAGCTGAGCTATTCCTGACGATGGACATGATGGACAAGCAGGGTATTGACCCTCAGGTTATTCTTGATGCCGACCGACTGACTGTTAAGCGGTCCAAagctgtctcttataca                           >  1:29588/1‑287 (MQ=255)`  
                                                                                                                                                                                                                                                                              `tACCTTCAAGCGGACGCTAAGGACGGAGAATTCCGTACAGCCATCGGAACCATGGT‑‑CACTGACGCGGGTCAAGAGTGGTCTGCCGCTGTGATTAACGGTAAGTTACAAGAACGAACCCAAGCTATGGATGCTCTGCGCAGAATCCGAAAGGCTGACCCTCAGTTGATTGCTTCGCTATACCCAGACCAAGCTGAGCTATTCCTGACGATGGACATGATGTACAAGCAGGGTATTGACCCTCAGGTTATTCTTGATGCCGACCGACTGACTGTTAAGCGGTCCAAAGAGCAACGCTTTGAgg                          <  1:674118/301‑1 (MQ=255)`  
                                                                                                                                                                                                                                                                                `ccTTCAAGCGGACTCTAAGGACGGAGAAGTCCGTAAAGCCATCGGAACCATGGT‑‑CACTGACGCTGGTCAAGAGTGGTCTGCCGCTGTGATTAACGGTAAGTAACCAGAACGAACCCCAGCTATGGATGCTCTGCGCAGAATCCGCAATGCTGACCCTCAGTTGATTGCTGCGCTATACCCAGACCAAGCTGAGCTATTCCTGACGATGGACATGATGGACAAGCAGGGTATTGACCCTCAGGTTATTCTTGATGCCGACCGACTGACTGTTAAGCGGTCCAAAGAGCAACGCTTTGAGgat                        <  1:428507/301‑1 (MQ=255)`  
                                                                                                                                                                                                                                                                                  `ttCAAGCGGACTCTAAGGACGGAGAATTCCGTACAGCCATCGGAACCATGGT‑‑CACTGACGCTGGTCAAGAGTGGTCTGCCGCTGTGATTAACGGTAAGTTACCAGAACGAACCCCAGCTATGGATGCTCTGCGCAGAATCCGCAATGCTGACCCTCAGTTGATTGCTGCGCTATACCCAGACCAAGCTGAGCTATTCCTGACGATGGACATGATGGACAAGCAGGGTATTGACCCTCAGGTTATTCTTGATGCTGACCGACTGACTGTTAAGCGGTCCAAagctgtctcttatacacatct                      >  1:476565/1‑282 (MQ=255)`  
                                                                                                                                                                                                                                                                                   `tCAAGCGGACGCTAAGGACGGAGAATTCCGTACAGCCATCGGAACCATGGT‑‑CACTGACGCTGGTCAAGAGTGGTCTGCCGCTGTGATTAACGGTAAGTTACCAGAACGAACCCCAGCTATGGATGCTCTGCGCAGAATCCGCAATGCTGACCCTCAGTTGATTGCTGCGCTATACCCAGACCAAGCTGAGCTATTCCTGACGATGGACATGATGGACAAGCAGGGTATTGACCCTCAGGTTATTCTTGATGCCGACCGACTGACTGTTAAGAGGTCCAAAGAGCAACGCTTTGAGgatgat                     <  1:643910/301‑1 (MQ=255)`  
                                                                                                                                                                                                                                                                                    `cAAGTGGACTCTAAGGACGGAGAATTCCGTACAGCCATCGGAACCATGGT‑‑CACTGACGCTGGTCAAGAGTGGTCTGCCGCTGTGATTAACGGTAAGTTACCAGAACGAACCCCAGCTATGGATGCTCTGCGCAGAATCCGCAATGCTGACCCTCAGTTGATTGCTGCGCTATACCCAGACCAAGCTGAGCTATTCCTGACGATGGACATGATGGACAAGCAGGGTATTGACCCTCAGGTTATTCTTGATGCCGACCGACTGACTGTTAAGCGGTccctgtctcttatacacatctccgagc                    >  1:21006/1‑276 (MQ=255)`  
                                                                                                                                                                                                                                                                                     `gaGCGGACTCTAAGGACGGAGAATTCCGTACAGCCATCGGAACCATGGT‑‑CACTGACGCTGGTCAAGAGTGGTCTTCCGCTGTGATTAACGGTAAGTTACCAGAACGAACCCCAGCTATGGATGCTCTGCGCAGAATCCGCAATGCTGACCCTCAGTTGATTGCTGCGCTATACCAAGACCAAGCTGAGCTATTCCTGACGATGGACATGATGGACAAGCAGGGTATTGACCCTCAGGTTATTCTTGATGCCGACCGACTGACTGTTAAGCGGTCCAAAGAGCAACGCTTTGAGGATGATaa                   <  1:186022/300‑1 (MQ=255)`  
                                                                                                                                                                                                                                                                                            `ctctAAGGGCGGAGCATTCCGTACAGCCATCGGAACCATGGT‑‑CACTGACGCTGGTCAAGAGTGGTCTGCCGCTGTGATTAACGGTAAGTTACCAGAACGAACCCCAGCTATGGATGCTCTGCGCAGAATCCGCAATGCTGACCCTCAGTTGATTGCTGCGCTATACCCAGACCAAGCTGAGCTATTCCTGACGATGGACATGATGGACAAGCAGGGTATTGACCCTCAGGTTATTCTTGATGCCGACCGACTGACTGTTAAGCGGTCCAAAGAGCAACGCTTTGAGGATGATAAAGCATTc            >  1:687722/1‑301 (MQ=255)`  
                                                                                                                                                                                                                                                                                                  `ggACGGAGAATTCCGTACAGCCATCGGAACCATGGT‑‑CACTGACGCTGGTCAAGAGTGGTCTGCCGCTGTGATTAAAGGTAAGTTACCAGAACGAACCCCAGCTATGGATGCTCTGCGCAGAATCCGCAATGCTGACACTCAGTTGATTGCTGCGCTATACCCAGACCAAGCTGAGCTATTCCTGAAGATGGACATGATGGACAAGCAGGGTATTGACCCTCAGGTTATTCTTGATGCCGACCGACTGACTGTTAAGCGGTCCAAAGAGCAACGCTTTGAGGATGATAAAGCATTCGAGTCt      <  1:370676/301‑1 (MQ=255)`  
                                                                                                                                                                                                                                                                                                  `ggACGGAGAATTCCGTACAGCCATCGGAACCATGGT‑‑CACTGACGCTGGTCAAGAGTGGTCTGACGCTGTGATTAACGGTAAGTTACCAGAACGAACCCAAGCTATGGATGCTCTGCGCAGAATCCGAAATGCTGACCCTCAGTTGATTGCTGCGCTATACCCCGACCAAGCTGAGCTATTCCTGACGCTGGACATGATGGACAAGCAGGGTATTGATCCTCAGGTTTTTCTTTATGCCGACCGACTGACTGTTAAGCGGTCAAAAGTGCAACGCCTTGGGGCTGATAAAGAATTCCATTCt      >  1:106128/1‑301 (MQ=255)`  

                                                                                                                                                                                                                                                                                                          `|`                                                                                                                                                                                                                                                                                                            
`GTTCAGAATCAGATGAACGCATGGACGAAAGCTCAGGCCAAGGCTCTGGACGATTCCATGAAGTCAATGAACAAACTTGACGTAATCGACAAGCAATTCCAGAAGCGAATCAACGGTGAGTGGGTCTCAACGGATTTTAAGGATATGCCAGTCAACGAGAACACTGGTGAGTTCAAGCATAGCGATATGGTTAACTACGCCAATAAGAAGCTCGCTGAGATTGACAGTATGGACATTCCAGACGGTGCCAAGGATGCTATGAAGTTGAAGTACCTTCAAGCGGACTCTAAGGACGGAGCATTCCGTACAGCCATCGGAACCATGGT‑‑CACTGACGCTGGTCAAGAGTGGTCTGCCGCTGTGATTAACGGTAAGTTACCAGAACGAACCCCAGCTATGGATGCTCTGCGCAGAATCCGCAATGCTGACCCTCAGTTGATTGCTGCGCTATACCCAGACCAAGCTGAGCTATTCCTGACGATGGACATGATGGACAAGCAGGGTATTGACCCTCAGGTTATTCTTGATGCCGACCGACTGACTGTTAAGCGGTCCAAAGAGCAACGCTTTGAGGATGATAAAGCATTCGAGTCTGCAC  >  NC_001604.1/29399‑29993`

  

Alignment Legend

Aligned base mismatch/match (shaded by quality score): `ATCG/ATCG` `< 3 ≤` `ATCG/ATCG` `< 18 ≤` `ATCG/ATCG` `< 29 ≤` `ATCG/ATCG` `< 37 ≤` `ATCG/ATCG` `< 38 ≤` `ATCG/ATCG`

Unaligned base: `atcg`    Masked matching base: `atcg`    Alignment gap: `‑`    Deleted base: `‑`
